# The Application of the Gibson Assembly Method in the Production of Two pKLS3 Vector-Derived Infectious Clones of Foot-and-Mouth Disease Virus

**DOI:** 10.3390/vaccines11061111

**Published:** 2023-06-18

**Authors:** Ploypailin Semkum, Nattarat Thangthamniyom, Penpitcha Chankeeree, Challika Keawborisuth, Sirin Theerawatanasirikul, Porntippa Lekcharoensuk

**Affiliations:** 1 Department of Microbiology and Immunology, Faculty of Veterinary Medicine, Kasetsart University, Bangkok 10900, Thailand; fvetpls@ku.ac.th (P.S.); golf_zealand@hotmail.com (N.T.); penpitchac56@gmail.com (P.C.); 2 Center for Advanced Studies in Agriculture and Food, KU Institute for Advanced Studies, Kasetsart University, Bangkok 10900, Thailand; 3 Virology and Cell Technology Research Team, National Center for Genetic Engineering and Biotechnology, National Science and Technology Development Agency, Pathum Thani 12120, Thailand; challika.kae@biotec.or.th; 4 Department of Anatomy, Faculty of Veterinary Medicine, Kasetsart University, Bangkok 10900, Thailand; fvetsrth@ku.ac.th

**Keywords:** Gibson assembly, foot-and-mouth disease virus, reverse genetics, vaccine, infectious clone

## Abstract

The construction of a full-length infectious clone, essential for molecular virological study and vaccine development, is quite a challenge for viruses with long genomes or possessing complex nucleotide sequence structures. Herein, we have constructed infectious clones of foot-and-mouth disease virus (FMDV) types O and A by joining each viral coding region with our pKLS3 vector in a single isothermal reaction using Gibson Assembly (GA). pKLS3 is a 4.3-kb FMDV minigenome. To achieve optimal conditions for the DNA joining, each FMDV coding sequence was divided into two overlapping fragments of approximately 3.8 and 3.2 kb, respectively. Both DNA fragments contain the introduced linker sequences for assembly with the linearized pKLS3 vector. FMDV infectious clones were produced upon directly transfecting the GA reaction into baby hamster kidney-21 (BHK-21) cells. After passing in BHK-21 cells, both rescued FMDVs (rO189 and rNP05) demonstrated growth kinetics and antigenicity similar to their parental viruses. Thus far, this is the first report on GA-derived, full-length infectious FMDV cDNA clones. This simple DNA assembly method and the FMDV minigenome would facilitate the construction of FMDV infectious clones and enable genetic manipulation for FMDV research and custom-made FMDV vaccine production.

## 1. Introduction

Foot-and-Mouth Disease (FMD) caused by the FMD virus (FMDV) is an important vesicular disease of livestock with a high economic impact [1]. FMDV is a positive-sense, single-stranded RNA virus belonging to the genus *Aphthovirus* in the family *Picornaviridae*. The genomic RNA of FMDV is approximately 8500 nucleotides and consists of a single open reading frame (ORF) coding for a polyprotein of approximately 2332 amino acids. The single coding region is flanked by long untranslated regions (UTRs) at 5′ and 3′ termini. FMDV 5′UTR contains complex secondary structures, which include an S fragment, a poly C tract, pseudoknots, a cis-acting replication element (cre) alternatively called 3B-uridylylation site (bus), and a cap-independent translation initiation site namely an internal ribosomal entry site (IRES), arranging from 5′ to 3′ direction [2]. The 3′UTR of FMDV has a poly-A tail of variable length and contains two stable stem loops involved in the negative-strand synthesis [3,4].

Reverse genetics is an essential technology for generating the cDNA clones of RNA viruses, which is a valuable tool for the studies on molecular mechanisms of viral infection and pathogenesis as well as the production of seed vaccine viruses. With the reverse genetics derived infectious cDNA clone, we can manipulate the viral genomes to create a seed vaccine virus with desirable properties such as decreased virulence, increased growth characteristics, and matched antigenicity with field viral strains [1]. Many studies reported successful establishments of reverse genetics systems based on T7 or SP6 RNA polymerase to drive the in vitro polymerization of the full-length infectious genomic RNA of FMDV using the infectious cDNA clones as the template [5,6,7,8]. Furthermore, RNA polymerase I (Pol I) [9] and RNA Pol I-Pol II reverse genetic systems [4,10] have been described for rescuing the genetically engineered FMDVs. These reverse genetics utilized the multistep subcloning strategy, which required non-viral and unique restriction enzyme recognition sites to obtain full-length infectious FMDV clones [4,5,9]. Searching for the appropriate restriction enzyme sites or introducing compatible sites to generate a recombinant FMDV genome is quite difficult. Generally, the conventional DNA cloning method, which employs restriction enzymes and ligase for DNA excision and assembly, is laborious and time-consuming. In addition, the DNA subcloning process may accumulate more mutations and produce scars at the vector-insert junction.

Recently, various in vitro DNA assembly procedures have been developed and become popular techniques to generate DNA libraries or perform gene cloning without restriction digestion and DNA ligation, such as the overlap extension PCR method [11], Golden gate method [12], and sequence-independent cloning method [13,14]. In addition, highly efficient DNA polymerases can process the whole viral genome with approximately 10–12 kb in length in an amplification reaction. Previously, the full-length cDNA of enterovirus 71, a picornavirus, was produced using the regular PCR method or fusion PCR technique and subsequently ligated into a plasmid vector [15]. However, the highly complex secondary structure of the 5′UTR and the long poly A tail are the most critical burden for the infectious clone production by PCR. In particular, in vitro polymerization of a long homopolymeric cytosine (C) sequence within the FMDV 5′UTR is highly strenuous, which is the major obstacle to obtaining the full-length cDNA clone.

The Gibson assembly (GA) method is a sequence-independent cloning that has been used widely for DNA construction due to its simple operation and comparatively low cost [16]. Construction of a plasmid with overlapping DNA fragments can be achieved in a single reaction without the DNA subcloning procedure by using the GA method. The GA reaction exploits the exonuclease to remove nucleotides in one strand from both ends of the DNA fragments in 5′ to 3′ direction and create single-stranded DNA overhangs for DNA annealing. Then, the gaps are filled by DNA polymerase, followed by covalently sealing the DNA ends with ligase [17]. Recently, GA cloning has been applied to generate genome-wide libraries for yeast surface display, in which 10 ^5^–10 ^7^ DNA fragments were seamlessly transferred to multiple vectors for a complete representation of a eukaryote genome [18]. This yeast surface display was used to identify protein–ligand interactions, such as drug or vaccine targets, ligand receptors, or protein-protein interactions. In addition, GA was found to be an efficient tool for transgene cloning in adenovirus vectors and producing adenovirus vector-derived infectious clones [19,20]. Moreover, the construction of the recent FDA-approved chimeric antigen receptor (CAR) T cells for cancer immunotherapy has been accelerated by PCR amplifying of nanobody sequences followed by GA cloning [21].

In this study, we have exploited the GA method to generate full-length FMDV infectious clones from two overlapping DNA fragments encompassing the whole coding sequence of FMDVs and assembled with a pKLS3 vector [22], in a single reaction. The pKLS3 is a DNA-based plasmid vector, invented by our group. It is an FMDV minigenome of approximately 4.3 kb in size and contains a T7 promoter to drive the transcription process. The key elements of pKLS3 comprise FMDV O189 5′UTR and 3′UTR followed by 48 adenine residues and hepatitis delta virus (hdv) ribozyme arranged in 5′ to 3′ direction with the introduced *Stu*I restriction site between the 5′ and 3′ UTRs for cloning. We placed the difficult PCR amplification regions in the pKLS3 as elements of the vector. Thus, only a full-length coding sequence is required to produce an infectious clone. Transfection of the full-length FMDV cDNA clones directly into BHK-21 cells yielded infectious viruses. The rescued FMDVs exhibited comparable viral growth characteristics and antigenic profiles to their parental viruses. Our study confirms that the full-length cDNA clones were successfully generated by the GA method, and they are the first GA-derived FMDV infectious clones thus far.

## 2. Materials and Methods

### 2.1. Cell Lines and Viruses

BHK-21 cells were maintained in Modified Eagle’s medium (MEM) (Gibco, New York, NY, USA) supplemented with 10% fetal bovine serum (FBS) (Gibco, New York, NY, USA), 2 mM L-glutamine, and 100 IU ampicillin per 1 mL media and incubated at 37 °C with 5% CO_2_. The viruses used in this study included FMDV type O isolate O/TAI/189/1987 (O189) provided by the Bureau of Veterinary Biology, DLD, Thailand, and type A isolate A/TAI/NP05/2017 (NP05) was isolated from a clinical sample collected from cattle during the 2017 outbreak in Thailand. Both viruses were cultivated in BHK-21 cells (ATCC, Manassas, VA, USA) maintained in MEM with 2% FBS. The viruses were harvested when the cytopathic effect (CPE) reached approximately 80% and stored at −80 °C until used.

### 2.2. Viral RNA Extraction and cDNA Synthesis

Parental viral RNAs and the rescued viral RNAs were extracted from the clarified viral suspensions using the Viral Nucleic Acid Extraction kit (GeneAid^®^, Taipei City, Taiwan) following the manufacturer’s protocol. RNAs from the rescued viruses were pre-treated with Ambion™ DNaseI (Thermo Scientific™, Boston, MA, USA) as suggested by the manufacturer’s instruction. The total RNAs were then used as templates for cDNA synthesis using Superscript III Reverse Transcriptase and random hexamers (Invitrogen™, Carlsbad, CA, USA). The cDNA synthesis was performed at 55 °C for 1 h, and the reaction was subsequently inactivated with RNase H by incubating at 70 °C for 15 min.

### 2.3. Primer Designs and Preparation of FMDV cDNA Fragments

To construct the full-length cDNA clone, a 6999 nucleotide-coding region of the FMDV genome was divided into two fragments of approximately 3–4 kb each, namely F1 and F2 for the 5′ and 3′ fragments, respectively. Each fragment contains two overlapping regions at both termini that could anneal with its neighbor fragments. The 5′-end of the primers NP05_F1_F and NP05_F2_R (Table 1) contains the overlapping sequences for DNA joining, while the sequences of the remaining primers comprise all overlapping fragments. The primers were manually designed using nucleotide sequences displayed by the SnapGene Viewer 5.3.2 (Dotmatics) to generate each DNA fragment for the GA technique. We started with the whole genome sequencing of both FMDV type A (NP05) and O (O189) and performed sequence analysis using MegAlign version 7.1.0 (DNAStar) and SnapGene Viewer 5.3.2 (Dotmatics) to design the fragments with equal size and select for appropriate overlapping regions with comparable annealing temperatures for the joining fragments.

**Figure 1:**
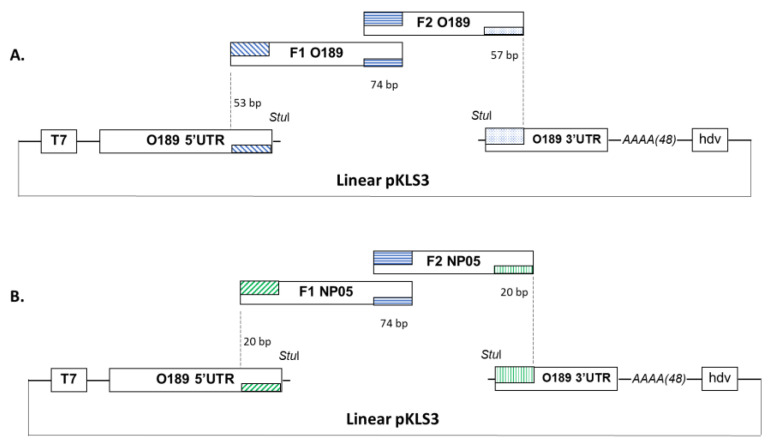
Construction of recombinant plasmids with FMDV cDNA clones. Plasmid pKLS3 comprises FMDV O189 5′UTR downstream of T7 promoter followed by *Stu*I restriction enzyme recognition site, FMDV 3′UTR, 48 adenine residues, and a ribozyme sequence of hepatitis delta virus. Three DNA fragments were designed for the GA reaction, in which FMDV 5′UTR at 3′ end of the linearized pKLS3 overlaps with 53 (O189) or 20 (NP05) bp at 5′ end of F1 fragment (~3.8 kb); 74 bp at 3′ end of F1 overlaps with 5′ end of F2 fragment (~3.2 kb); and 57 (O189) or 20 (NP05) bp at 3′ end of fragment F2 overlaps with 5′ end of FMDV 3′UTR within the linearized pKLS3. (**A**) Plasmid pKLS3 with FMDV type O, O189, coding sequence. (**B**) Plasmid pKLS3 with FMDV type A, NP05, coding sequence.

To generate an O189 infectious clone, cDNA of FMDV O189 was used as the template to amplify O189 fragment 1 (O189 F1) and fragment 2 (O189 F2) with primers OF1_F/F1_R and F2_F/OF2_R, respectively. The DNA assembly products were examined by gel electrophoresis. Three overlapping regions were designed for the DNA joining as described previously (Figure 1A). Firstly, the 53 bp of FMDV 5′UTR sequence at 3′ end of the linearized pKLS3 vector [22] (Patent Application Number: 1901006625) overlaps with 5′ end of the O189 F1; secondly, 74 bp of the O189 F1 3′ terminus overlaps with 5′ end of the O189 F2 (2C region); and finally, 57 bp of O189 F2 3′ terminus overlaps with 5′ end of FMDV 3′UTR sequence within the pKLS3. The PCR reactions were carried out using Phusion™ high-fidelity DNA polymerase (Thermo Scientific™, Boston, MA, USA) according to the manufacturer’s protocol. O189 F1 and F2 PCR products were purified and subsequently subcloned into pGEM-T easy vector (Promega, Madison, WI, USA) resulting in pOF1 and pOF2, respectively. The nucleotide sequences of both fragments were verified by Sanger sequencing (Macrogen, Seoul, Republic of Korea). The clones with correct nucleotide sequences were then used as templates to prepare O189 F1 and F2 fragments for the GA reaction.

The full-length coding region of FMDV type A, NP05 (accession number: OQ745961), was created similarly to the method described for O189. NP05 fragments 1 (NP05 F1) and 2 (NP05 F2) were amplified using primers NP05_F1_F/ F1_R and F2_F/NP05_F2_R (Table 1), respectively. Similar to the O189 DNA fragment assembly, the three DNA joining regions for NP05 coding sequence and vector assembly included 20 bp of FMDV O189 5′UTR sequence at 3′ end of the linearized pKLS3 vector overlapping with the 5′ end of NP05 F1, 74 bp of NP05 F1 3′ terminus overlapping with 5′ end of the NP05 F2 (2C region), and 20 bp of NP05 F2 3′ terminus overlapping with FMDV O189 3′UTR sequence at 5′ end of the pKLS3 (Figure 1B).

### 2.4. Gibson Assembly Reactions

The Gibson assembly (GA) reaction was used to assemble two FMDV fragments to the pKLS3 vector [22] to generate an infectious clone. The pKLS3 vector was linearized with *Stu*I (New England BioLabs, Ipswich, MA, USA) and then purified using the HiYield™ Gel/PCR DNA Fragments Extraction Kit (RBC Bio-science, Taipei City, Taiwan). O189 F1 and F2 fragments prepared from the previous step were also purified similarly. Then, O189 F1, O189 F2, and the linearized pKLS3 vector were assembled in an isothermal GA reaction (Gibson Assembly^®^ Cloning Master Mix) (New England BioLabs, Ipswich, MA, USA). following an optimized protocol based on the manufacturer’s suggestion (New England BioLabs, Ipswich, MA, USA). Briefly, 25 ng each of DNA fragments (linearized pKLS3, O189 F1 and O189 F2) was mixed with 10 μL of 2X Gibson Assembly Master mixture in a 20 µL reaction and subsequently incubated at 50 °C for 1 h in a thermocycler. The assembled DNA fragments were examined by electrophoresis through 0.8% agarose gel (Cambrex Bio Science, Rockland, MA, USA) in 1X TAE buffer (Sigma-Aldrich, St. Louis, MO, USA). In addition, the GA reaction of FMDV type A (NP05) was also performed to combine the linearized pKLS3 with NP05 F1 and F2 using the Gibson Assembly kit and method as described above.

### 2.5. Electro-Transformation

For electro-transformation, the GA reaction was diluted in three volumes of DNase and RNase-free dH2O (Thermo Scientific™, MA, USA) prior to starting the electroporation process. A volume of 2 microliters of the diluted GA reaction was transferred into a pre-cool cuvette (1 mm, Bio-Rad, Hercules, CA, USA) containing 50 µL of electrocompetent *E. coli*, BL21. The electroporation was performed using GenePulser^®^ (Bio-Rad, CA, USA) with the parameter set at 1700 kV, 25 µF, 200 Ω. Then, 950 µL of SOC medium (New England BioLabs, MA, USA) was immediately added into the transformant cuvette, which was further incubated at 37 °C for 16–18 h. A volume of 150 microliters of the transformants was plated onto LB agar plates containing 100 mg/mL ampicillin. The plates were incubated at 26 °C for 36–38 h. The positive colonies were selected for further culturing in LB broth. Both pKLS3_O189 and pKLS3_NP05 were isolated from the *E. coli* culture using GeneMark^®^ Plasmid Minipreps kit following the manufacturer’s protocol (GeneMark, Taipei City, Taiwan). The plasmids were submitted for nucleotide sequencing (Macrogen, Seoul, Republic of Korea).

### 2.6. Transfection of Infectious Clones and Virus Recovery

For transfection, BHK-21 cells were cultured in a 6-well plate overnight and reached 70–80% confluent when used. The recombinant infectious plasmids, pKLS3_O189 and pKLS3_NP05, were transfected into the BHK-21 cells together with pCAGGS_T7 and pCAGGS_P3, so-called the tri-transfection method [22], using Lipofectamine^®^ 2000 (Invitrogen, USA). Briefly, 2 µg pKLS3_O189 or pKLS3_NP05, 6 µg pCAGGS_T7, and 2 µg pCAGGS_P3 were added in a tube containing 250 µL Opti-MEM^®^ reduced serum medium (ThermoFisher Scientific, MA, USA) and mixed well. In another tube, 25 µL Lipofectamine^®^ 2000 (Invitrogen, CA, USA) was also diluted in 250 µL Opti-MEM^®^ and mixed well. The diluted plasmids were transferred into the diluted transfection reagent and mixed gently before incubating for 20 min at room temperature. To prepare for transfection, the BHK-21 cells were washed once with the Opti-MEM^®^. All traces of media were removed and replaced with 2 mL of Opti-MEM^®^ before adding the mixture of transfection reagent and DNA complexes onto the cell monolayer in a dropwise manner. The cells were incubated at 34 °C with 5% CO_2_. A negative control well (co-transfecting with pCAGGS_T7 and pCAGGS_P3) and a positive control well (tri-transfecting with pKLS3_GFP, pCAGGS_T7, and pCAGGS_P3) were included in the experiment. After 48 h post-transfection (hpt) or when cytopathic effect (CPE) reached 80–90%, cells were frozen at −80 °C and thawed once. The cell suspension containing the rescued virus (either rO189 or rNP05) was clarified by centrifugation at 6000× *g* at 4 °C for 10 min. The clarified fluid containing the rescued viruses, passage 0 (p0), was collected and consecutively sub-passaged in BHK-21 cells 13 times (p1-13) to increase virus yields. The rescued viruses were detected by RT-PCR using DNaseI-treated RNAs as templates with FMDV 2B and type-specific VP1 primers (Table 2). The PCR products were verified by DNA sequencing (Macrogen, Seoul, Republic of Korea).

### 2.7. Detection of the Rescued FMDVs by RT-qPCR

Total RNAs were isolated from 200 µL of the supernatant containing the rescued viruses from each passage. A hundred nanograms of the RNAs were used as the template for cDNA synthesis using random hexamers. Subsequently, quantitative PCR (qPCR) was performed as described previously [23] using forward (FMDV 5′UTR_F) and reverse (FMDV 5′UTR_R) primers (Table 2), which target a conserved sequence in the internal ribosomal entry site (IRES) within the 5′UTR. A plasmid containing the conserved sequences of FMDV IRES was ten-fold serially diluted from 1 to 0.000001 ng (2.87 × 10^2^ and 2.87 × 10^8^ DNA copy numbers) and used as templates for amplification to generate a standard curve. The 10 µL qPCR reaction comprised SsoFast™ EvaGreen^®^ Supermix (2X) (Bio-Rad, CA, USA), 5 pM/µL of each primer, and 4 µL of the cDNA template. The qPCR reaction was performed in the CFX96 Touch real-time system (Bio-Rad, USA) using the following thermal profile: 95 °C for 30 s, followed by 40 cycles of 95 °C for 5 s, 60 °C for 5 s and 65 °C for 10 s, and a final reaction at 95 °C for 5 s. Both DNA standards and samples were run in duplicate. The results were analyzed using CFX Maestro™ software (Bio-Rad, USA), and a quantification cycle (Cq) value was assigned to each reaction. The absolute quantification of viral copy numbers (copies/µL) of the rescued and their parental viruses were calculated automatically based on the standard curve by the software.

### 2.8. Growth Characterization of the Rescued FMDVs in BHK-21 Cells

To perform viral growth characterization, viral titers of the wild type and rescued viruses, rO189 at passage 10 and rNP05 at passage 13, were determined in BHK-21 cells as described previously [24]. Briefly, the BHK-21 cells were seeded in a 6-well plate and incubated at 37 °C overnight. On the next day, the cells were washed once with MEM before being inoculated with the rescued (rO189 and rNP05) and their parental viruses (type O, O189 and type A, NP05) at an MOI of 0.01. After an hour of adsorption, all traces of viruses and media were removed and replaced with 2 mL of the maintenance medium containing MEM with 2% FBS and 1% Antibiotic-Antimycotic (Thermo Fisher Scientific, MA, USA). The infected cells were incubated at 34 °C for 24 h. Two hundred and fifty microliters of the supernatants were collected from each well at different time points at 4 h intervals until 24 h. The supernatant was kept at −80 °C until used. The virus titers at each time point were determined as 50% tissue culture infective dose (TCID50/mL) following Reed and Muench method (Reed and Muench, 1938).

### 2.9. Immunoperoxidase Monolayer Assay (IPMA)

To confirm the antigenicity of the rescued viruses, IPMA was performed as described previously [24]. Briefly, BHK-21 cells overnight grown in a 96-well plate were inoculated with the rescued and parental FMDV types A and O at an MOI of 0.1. After 24-h incubation at 37 °C with 5% CO_2_, the infected cells were fixed with absolute methanol for 30 min and washed with 0.5% PBST (PBS containing 0.5% Tween 20) three times. Then, the cells were incubated with an affinity-purified monoclonal antibody (mAb) specific to FMDV VP2, 3E11, as the primary antibody (in-house preparation) at a dilution of 1:50 for 1 h. The excess antibody was washed with 0.5% PBST three times before incubating with HRP-conjugated goat anti-mouse IgG (Sigma-Aldrich, St. Louis, USA) as the secondary antibody at a dilution of 1:500 for 1 h. The cells were washed with 0.5% PBST three times before incubating with the substrate for 10–15 min at room temperature. The brown-staining positive cells were observed using an inverted microscope (Olympus^®^, Tokyo, Japan).

To demonstrate the specific reactivity between the wild type (WT) and rescued FMDVs with the mAb 3E11, isotype controls were performed using porcine circovirus type 2 (PCV2) ISU31 [25] and its cognate mAb 12G3 [26]. The PCV2 was inoculated onto overnight grown PK15 cells in a 96-well plate at 10^4^ TCID50 per well and non-infected PK-15 cells were included as the mAbs 12G3 and 3C11 background control. IPMA was performed as described above using mAbs 12G3 and 3C11 as the primary antibodies.

### 2.10. Plaque Assay

The ability of rescued FMDVs to form plaque was assessed in BHK-21 cells as previously described [27]. Briefly, BHK-21 cells were grown in 6-well plates to reach 80% confluence. Cells were inoculated with the rescued and parental FMDV types A and O. After 1 h of incubation, all traces of inoculums were removed, and the cells were overlaid with 2 mL of melting agarose (Cambrex Bio Science, Rockland, CA, USA) in the maintenance medium. Once the agarose became solidified, the culture plate was incubated at 37 °C with 5% CO_2_ for 24 h. The cells were fixed with 4% formaldehyde, and the agar was removed. Then the cells were stained with Crystal violet (Biotechnical Co., Ltd., Bangkok, Thailand) for 10 min followed by rinsing with plenty of distilled water. The clear plaque was observed and imaged over a white-light box.

## 3. Results

### 3.1. Construction of the GA-Derived FMDV Infectious Clones

**Figure 2:**
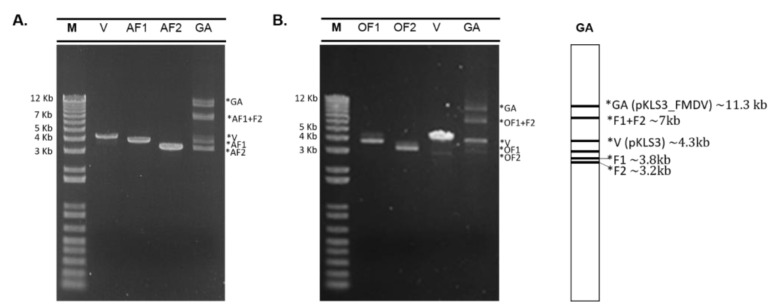
Electrophoretic photographs of two GA reactions. Input DNA fragments in each GA reaction include 4.3 kb of the linearized pKLS3 vector (V), 5′coding sequence of FMDV fragment 1 (AF1 or OF1), and 3′coding sequence of FMDV fragment 2 (AF2 or OF2). (**A**) FMDV type A, NP05, composes of 3.8 kb AF1 and 3.3 kb AF2. (**B**) FMDV type O, O198, comprises 3.8 kb OF1 and 3.3 kb OF2. The DNA fragments in each GA reaction (lane GA) compose of ~11.3 kb of pKLS3 plus the full-length FMDV coding region (GA), ~7 kb of assembled F1 and F2 or pKLS3 and F1/F2, ~3.8 kb of F1 and ~3.2 kb of F2, respectively.

By DNA sequence analysis, the O189 F1 comprises 3885 nucleotides of P1-2A-2B-2C regions, and O189 F2 contains 3297 nucleotides encompassing 2C-P3 regions (Figure 1A), while those of NP05 F1 and NP05 F2 are 3853 and 3260 nucleotides, respectively (Figure 1B). In the GA reactions, FMDV F1 and F2 were assembled with the linearized pKLS3 to generate recombinant infectious clones. As a result, both O189 and NP05 GA reactions composed of mixed DNA components including pKLS3 with full-length FMDV genome (~11.3 kb), incomplete assembled sequences of F1 plus F2 or F1/F2 bound with the linearized pKLS3 (~7 kb), and a single fragment of pKLS3 (~4.3 kb), F1 (~3.8 kb), or F2 (~3.2 kb) (Figure 2). The key successes of the assembly reaction to obtain the infectious clone were the optimal length of each DNA fragment and the overlapping design. Annealing temperatures for all joining regions and the concentration of each fragment should be uniform. Upon transformation of the GA reaction into *E. coli*, the plasmid DNA clones were selected, and integrities of the 11.3 kb DNA sequences were verified by sequencing. The DNA clones containing corrected nucleotide sequences of the full-length coding region of FMDV types O and A were named pKLS3_O189 and pKLS3_NP05, respectively. The results confirmed that pKLS3 could carry either homologous, FMDV type O, or heterologous, FMDV type A, gene.

### 3.2. Rescue of FMDV Infectious Clones

**Figure 3:**
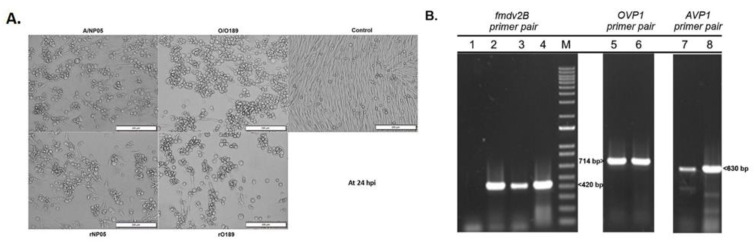
Cytopathic effect (CPE) and RT-PCR products of the rescued and parental FMDVs at 24 h post-infection in BHK-21 cells. (**A**) The rescued FMDVs of both types A (NP05) and O (O198) produced CPE similarly to the corresponding parental viruses while mock-infected cells (control) did not develop CPE. (**B**) Electrophoretic photographs demonstrated the RT-PCR products amplified with common FMDV (FMDV 2B) and type-specific (VP1) primers. The FMDV 2B primers produced an approximately 420 bp DNA fragment, while the FMDV type-specific primers amplified the VP1 gene region and generated 630 and 714 bp DNA fragments for rNP05 and rO189, respectively. Lanes 1–4 are RT-PCR products amplified with FMDV 2B primers; control [1], rescued O189 [2], rescued NP05 [3], and wild-type FMDV [4]. Lanes 5 and 6 are PCR products amplified using cDNA from the rescued and parental O189 viruses, respectively, with OVP1 primers. Lanes 7 and 8 are PCR products amplified with AVP1 primers using cDNA from the rescued and parental NP05 viruses, respectively.

To rescue the cDNA-derived infectious FMDV clones, the clarified supernatant from the transfected BHK-21cells containing the rescued O189 (rO189) and rescued NP05 (rNP05) passage 0 (p0) were consecutively passed onto the overnight seeded BHK-21 cells to increase the viral yields. From passage 5 onward, rO189 grew efficiently in BHK-21 cells and developed CPE rapidly, which reached 80–90% by 20 hpi (Figure 3A). On the other hand, rNP05 had to be passed in BHK-21 cells 13 times to develop the rapid CPE-producing characteristic with complete cell lysis by 24 hpi. The rescued viruses in each passage were detected by RT-PCR using both FMDV 2B and typing-specific primers. The results of rO189 and rNP05 RT-PCRs showed strong DNA bands with expected sizes, similar to those of their parental viruses (Figure 3B). These results indicate that both pKLS3_O189 and pKLS3_NP05 were infectious clones that could infect and replicate in BHK-21 cells like their parental viruses.

### 3.3. Quantification of the Rescued FMDV by RT-qPCR

**Figure 4:**
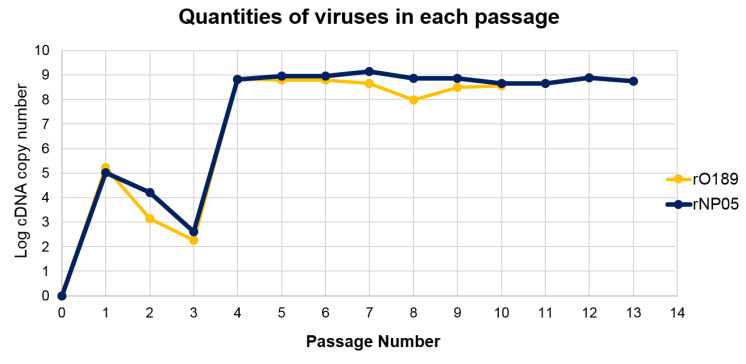
A chart of the plot between logarithmic based 10 of cDNA copy numbers of the rescued FMDVs and passage numbers. Passage 0 (p0) was supernatant harvested from transfected cells and media. p1-13 were the supernatants that were passed in BHK-21 cells 1–13 times, respectively. The rescued FMDV cDNAs were determined by RT-qPCR using the absolute quantification method. The cDNA copy numbers were calculated based on the mean Cq values.

To estimate the number of rescued viruses in each passage, we performed RT-qPCR using the absolute quantification method. The cDNA copy number was calculated based on the Cq values and transformed to logarithmic based 10 (log). A standard curve was plotted between the Cq values as the y-axis, and the log initial copy numbers of the cDNA templates (log 2–9) was plotted as the x-axis. A linear regression equation was fitted, which demonstrated a high correlation of the Cq values and template copy numbers with R^2^ = 0.99. Analyses of the melting curves from both standards and tested samples demonstrated a single peak of temperatures ranging from 85.5 to 87 °C. The cDNA copy numbers of the rescued viruses were plotted against the corresponding passage numbers (Figure 4). The results showed that rO189 was detected in the transfected cells starting from p1 until the last passage examined. However, the amount of cDNA decreased shortly during p2-3 before abruptly increasing from p4-10 with the average cDNA copy number of 4.5 × 10^8^, which was higher than 1.38 × 10^7^ copies of the parental virus, O189. Likewise, the rNP05 virus was noticeable in p1, but the cDNA copy number was lower during p2-3 before suddenly raised up from p4-13 with the average copy number of 7.5 × 10^8^, which was slightly lower than 9.87 × 10^8^ copies of the parental virus, NP05. Although the cDNA significantly increased from p4 onward, the CPE was first noticed at p5 for O189 and p13 for NP05. The results indicated that the rescued viruses have been generated since p1 and stably persisted in the culture. However, there was a lag time for the existing high-titer populations to become dominant and develop a detectable CPE.

### 3.4. Biological Characterizations of the Rescued FMDVs

**Figure 5:**
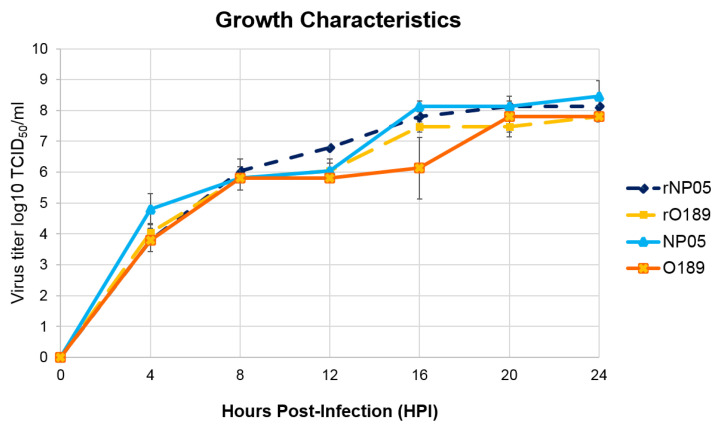
The chart demonstrated the growth kinetics of the rescued and parental FMDVs. The rescued and their parental FMDV types O (O189) and A (NP05) were inoculated onto BHK-21 cells at 0.01 MOI and the supernatants were collected at four-hour intervals (0–24 hpi). Viral titers were transformed to logarithmic-based 10, which were plotted against collecting times in hours post inoculation (hpi). The growth characteristics of the rescued (rNP05 and rO189) and their parental viruses (NP05: A/TAI/NP05/2017 and O189: O/TAI/189/1987) were not significantly different.

The growth characteristics of the rescued viruses were determined and compared with their parental strains, O189 (type O) and NP05 (type A). Both rescued and their parental viruses showed similar growth properties in the BHK-21 cells. The CPE was first recognized in all viruses at 4 hpi, in which approximately 30% of cell death were observed. The CPE gradually increased until it reached more than 80% at 20 hpi. Titers of rNP05 and the parental viruses were similar with the mean titers of 10^8.5^ TCID50/mL at 20 hpi, which was slightly higher than those of rO189 and its cognate wild-type virus (the mean titers = 10^7.8^ TCID50/mL) at the same time point. The titers of the rescued viruses, rO189 and rNP05, and the corresponding parental strains in BHK21 cells were not significantly different at each time point. In addition, the highest titers of rNP05, rO189, and O189 were at 20 hpi (Figure 5). However, the titer of the wild-type virus, NP05, peaked at 24 hpi. The results indicated that both rescued and the parental viruses had similar replication rates and grew to high titers in BHK-21 cells.

**Figure 6:**
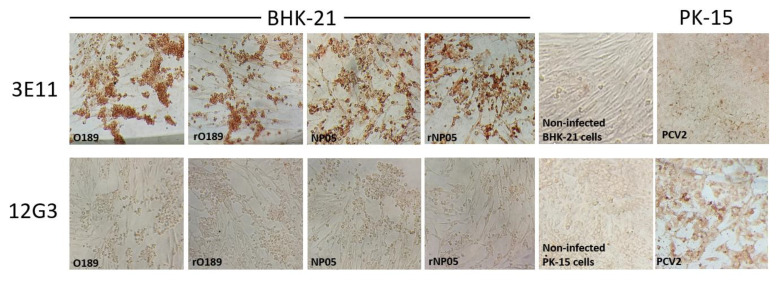
Immunoperoxidase monolayer assay (IPMA) demonstrates specific reactivity between wild-type (WT) or rescued FMDVs and a monoclonal antibody (mAb) raised against FMDV (3E11). FMDV types O (O189) and A (NP05), and rescued virus types O (rO198) and A (rNP05) infected BHK-21 cells reacted strongly with the mAb 3E11 as shown by the brown color. mAb 3E11 did not bind to non-infected BHK-21 cells and porcine circovirus type 2 (PCV2) infected PK-15 cells, which served as 3E11 background and isotype controls, respectively. Both WT and rescued FMDVs did not react to mAb 12G3, a mAb specific to PCV2 [26]. PCV2-infected PK-15 cells show a positive signal with mAb 12G3, while non-infected PK-15 cells remained negative. (Magnification of 100×).

To examine viral antigenicity, IPMA was performed on BHK-21 cells infected with the rescued and their parental viruses for 24 h using a monoclonal antibody specific to FMDV VP2, 3E11, as the primary antibody. This monoclonal antibody could bind to antigens from both FMDV types O and A. The positive reactivity appeared as brown staining in the cytoplasm of infected cells during the late phase of the virus life cycle. The results showed that both rO189 and rNP05 could react with the monoclonal antibody similar to their parental viruses (Figure 6). Reactivities between non-infected BHK-21 cells with mAb 3E11 (background control) and PCV2-infected PK-15 cells with 3E11 (isotype control) were negative. These PCV2-infected cells reacted strongly with their cognate PCV2-specific mAb 12G3 [25,26].

**Figure 7:**
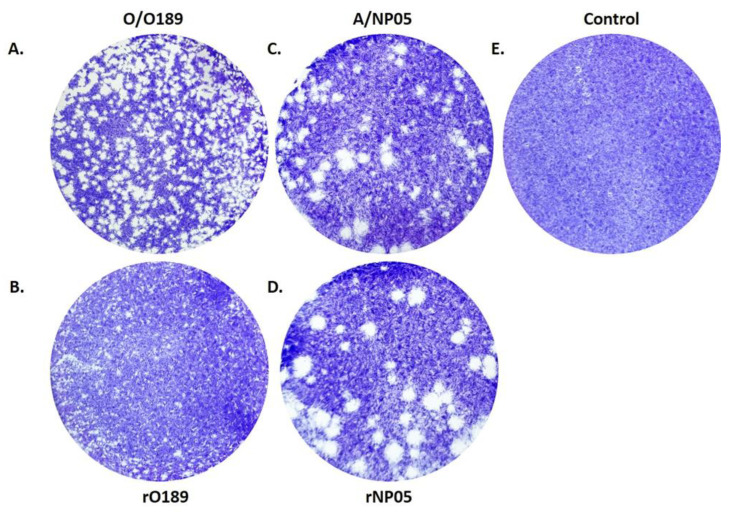
Plaque Morphologies of wild-type O189 (**A**), rescued O189 (**B**), wild-type NP05 (**C**), and rescued NP05 (**D**). The plaques were observed at 24 h post-infection. Non-infected BHK-21 cells were also included as a negative control (**E**).

Plaque formation by each virus was examined in the monolayer of BHK-21 cells. The plaque morphologies were visualized by staining with a crystal violet solution. The wild-type O189 produced a significantly smaller plaque than the wild-type A/NP05 (Figure 7) as observed at 24 hpi. Both rO189 and rNP05 also formed plaques with significantly different sizes, however, similar morphology to their parental viruses.

## 4. Discussion

Reverse genetics is an essential technique to generate infectious cDNA clones for RNA viruses, which is a valuable platform for genetic manipulations in the molecular study on virus biology and mechanisms of viral infection and pathogenesis. Furthermore, it is a vital tool for creating vaccine virus candidates with the desired biological properties. Generally, the construction of an infectious clone requires laborious subcloning steps. In this study, we bypassed the DNA subcloning process and generated FMDV infectious clones by a single reaction of DNA fragment assembly using the GA method. We found that GA was a simple and reliable DNA joining method that could facilitate the construction of FMDV infectious clones. However, we found some strenuous steps worth addressing in the following discussion.

The FDMV UTRs are cis-acting elements essential for viral replication, transcription, translation, and infectivity. We found that the generation of the complete 5′UTR fragment was the most challenging step of the infectious clone construction. The 1100 nt 5′UTR is composed of 371 nucleotide SF, a poly C tract, and 685 nucleotide LF arranged in a 5′ to 3′ direction, which shows that amplifying the whole 5′UTR in one reaction to maintain the sufficient length of poly C tract required for the viral life cycle was quite difficult. A previous study reported that natural FMDVs contained 100 to 420 cytosine residues in the poly C tracts, and the poly C length was associated with viral virulence [28]. However, to generate a genetically engineered FMDV, only a minimum length of poly C tract is required to maintain viral infectivity. The first described full-length infectious clone of FMDV in 1990 [8] verified that the lowest number of cytosines in the poly C tract required for infectivity of the plasmid-derived RNA was 32 residues. In the later study, poly C tracts of the infectious cDNA clones derived from FMDV type A12 contained 6–35 cytosine residues [7], while the recent study reported the full-length infectious cDNA clone of FMDV with 17 cytosine residues [29]. We found that 14 cytosine residues in the poly C tract of pKLS3 were sufficient for the generation of the infectious clones.

In addition to the poly C tract, the minimum length of the poly-A tail also plays an important role in the infectivity of FMDV [30]. A study on the significance of the poly-A in an FMDV sibling virus, Seneca virus A (SVA), revealed that the minimum length of the poly-A tail in the competent replicon was 14 adenine residues [31]. However, the number of adenines was increased upon consecutively passage of the rescued progeny viruses in BRS T7/5 cells. We also experienced that the length of both the poly-C tract and poly-A tail were crucial factors for successful virus rescues. Our pKLS3 contains 14 cytosine residues in the poly-C tract within 5′UTR and 48 adenine residues of the poly-A tail. The clones with smaller cytosine or adenine residues were not infectious. In addition, when the rescued FMDVs were serially passaged in cell cultures, the poly-C and poly-A tail lengths were still retained. Sequence analysis revealed that upon passaging in BHK-21 cells, some nucleotide substitutions occur in the 5′UTR of the final rescued FMDV of both types A and O. However, their secondary structures predicted using the UNAfold server (RNA Folding Form V2.3) were not significantly different from the infectious plasmid. Moreover, alignment of O189 5′UTR of the minigenome, pKLS3, and other FMDVs including type ASIA 1 (Asia 1 IND 63/72), type A (isolate a28 Turkey iso44) and O (isolate o5india iso34) showed more than 90% identity, but their secondary structures are maintained. Especially, the cis-acting elements formed by stem-loop structures that are involved in the biological activities of the viruses, such as pseudoknots (PKs), cre, and IRES, are conserved. The collective results assumed the potential compatibility of the minigenome backbone with the other FMDV types.

Another challenge of plasmid DNA-based FMDV reverse genetics development is the generation of rescued viruses from the full-length infectious RNAs. It has been shown that some FMDV proteins including non-structural proteins 3A, 3B, 3C, and 3D could be supplied in trans to enhance the replication of the infectious clones [32]. Co-transfection of the 3A together with the reverse genetic plasmid could restore the replication activity of a replication-defective FMDV replicon. Furthermore, the 3D protein that was supplied in trans could efficiently interact with cis-acting elements within the FMDV 5′UTR. Moreover, the 3AB proteins overexpressed in FMDV-infected cells were uridylylated, which could initiate viral transcription and contribute to increased FMDV replication [33]. We assumed that these trans-activator proteins could assist the polymerization of the genomic and anti-genomic RNAs once the first RNA strand was generated by T7 RNA polymerase. Therefore, we have selected FMDV P3 as the trans-activator for the replication and transcription of the infectious clone. The trans-activator function of pCAGGS_P3 was also evaluated as a helper plasmid in the tri-transfection experiment [22].

Upon monitoring every passage of the rescued FMDV O189 and A/NP05, we could detect the viral nucleic acids from the first passage onward and the detectable nucleic acid copy numbers were more than 10^8^ from passages 4–13, while the CPE was delayed until passages 5 and 13 for types O and A, respectively. This discrepancy between the delayed CPE formation and copy numbers of the rescued viral nucleic acids in the RT qPCR might come from interfering with the transfected pKLS3. In Figure 4, we used primers specific for 5′UTR of FMDV for the real-time PCR. The 5′ UTR is also present in the pKLS3 vector. Transfection with one plasmid (pKLS3 alone) into the BHK21 cells is usually more efficient than that with three plasmids (pKLS3, pT7 pol, and pP3) in a cell, required for the generation of an infectious virus. Upon transfection into the cells, pKLS3 might have been rejoined and could be transcribed by T7 RNA polymerase, which may be more efficient than the pKLS3 with the 7 kb insert. 3B is also present in pP3; however, it cannot replicate in mammalian cells. VP1 is present only in the FMDV coding sequences of the infectious clones. Therefore, it is possible that the 5′UTR observed in the earlier passages may be pKLS3 plus the rescued viruses. In addition, a previous study has shown that defective viruses containing deletion(s) in L or capsid coding regions were generated upon virus adaptation in cell culture [34]. These defective interfering viruses are composed of three classes including deletion of 417 nucleotides within the L region and deletions of 999 and 1017 nucleotides within the capsid coding sequence. BHK-21 cells transfecting with transcripts derived from the defective genomes showed no CPE production during 3–5 days of incubation. These defective RNAs could be infectious by complementing each other and producing CPE of more than 90% on day 5 after transfection. Most viral populations were mixtures of defective viruses; however, 1 out of 42 plaques contained viruses with complete genomic RNAs. The complementation most likely occurs from genetic recombination among the wild-type and the defective genomes because of strand switching during negative-strand synthesis [35]. In our study, transfection with pKLS3_O189 or pKLS3_NP05 might produce both RT-PCR-detectable defective and complete genomic RNAs. The majority of populations might be defective viruses, while a few viruses with complete genomes replicated slower. Therefore, the small number of virions with full-length genomes might need several days of accumulation to produce detectable CPE.

GA cloning has been used to construct infectious clones of many positive-sense, single-stranded RNA viruses, such as Dengue virus [36,37], West Nile virus [38], porcine reproductive and respiratory syndrome virus [39], and coxsackievirus A6 (CA6) [40]. The full-length cDNA clone of coxackievirus A6 was constructed from two overlapping fragments of 7.4 and 5 kb corresponding to the full-length coxackievirus A6 cDNA and the pSVA plasmid vector, respectively [40]. The first fragment contained the whole viral genome flanked by the T7 promoter at the 5′ end and 19 thymidine residues of poly T at the 3′ end, which was simply assembled with the neighboring fragment in a single GA reaction. Indeed, the coxackievirus A6 5′UTR contains about 744 nucleotides, whereas its 3′UTR region is around 83 nucleotides plus 27 adenine residues of the poly-A tail (GenBank accession no. KR706309). In our study, we placed the FMDV regulatory elements in the pKLS3 vector for the convenience of the subsequent GA cloning. Furthermore, the key success of the GA reaction was the comparable length of each joining DNA fragment. In this study, the 7 kb of the FMDV coding region was divided into two fragments of 3.3 and 3.8 kb, which were assembled into the 4.3 kb of the pKLS3 vector.

The first FMDV infectious cDNA clone invented by Zibert and colleagues was constructed by sticky-end ligation of DNA fragments to create a complete FMDV (strain O1K) cDNA in an in vitro transcribing vector [8]. The full-length viral RNA was produced in vitro, and the rescued viruses were generated by RNA transfection. Later, Bai XW et al. [30] also used restriction enzyme digestion and ligation of DNA fragments to construct full-length infectious cDNA clones. However, they were transfected with two plasmid DNA, one containing an infectious cDNA clone and another carrying T7 RNA polymerase. In both studies, searching for suitable single-cut restriction enzymes, and multiple restriction digestions, subcloning, and sequencings was required for the generation of the complete cDNA clones with the genome length. This laborious DNA subcloning method is time-consuming and may increase the accumulation of mutations. In our study, the GA reaction was applied in the DNA joining step in substitution of multiple subcloning, and sequencing procedures as the multiple DNA fragments (linearized pKLS3 and FMDV DNA fragments) could be connected in a single reaction. We also bypassed the DNA and plasmid purification steps because the GA reaction could be used to transform bacterial hosts directly. In our experience, this technique required fewer steps and reagents for cloning and eliminated the restriction enzyme digestion step. In addition, with the GA cloning, we could design the DNA joining region so that no retaining undesired nucleotides or scar sequences between the vector and insert fragments occurred in traditional cloning.

## 5. Conclusions

To our knowledge, this is the first report on infectious FMDV cDNA clones generated by the GA method, which could facilitate genetic manipulation for the studies on the molecular biology of viruses as well as the mechanisms of viral infection and pathogenesis. Furthermore, this simple method could be used to generate a custom-made FMDV for vaccine production. In addition to the GA-derived full-length cDNA, the indispensable component of our infectious clones is pKLS3, which contains all FMDV cis-acting elements essential for the biological activities of the viruses. It is an efficient platform for generating infectious cDNA clones of FMDV types O and A.

## Figures and Tables

**Table 1:** Oligonucleotide primers used to amplify FMDV cDNA fragments for GA cloning.

Primers	Sequences (5′–3′)
OF1_F	TGGATAGGCGACCGGAGGCCG
F1_R	AAGTCCTTGCCRTCAGGGTTCTGG
F2_F	CACTTYGACGGTTACAACCA
OF2_R	TCCTACGGCGTCGCTCGC
NP05_F1_F	TGATTGCCACTAAATTCAGGATGGACACAACTGACTGCTTTATCG
NP05_F2_R	TTGTGACATCTGAGGGAAGGTTATGCGTCACCACACAC

**Table 2:** Oligonucleotides for detection and quantification of the rescued FMDVs.

Primers	Sequences (5′–3′)
AVP1_F	ATGGATCCACCACCGCCACC
AVP1 R	ATAAGCTTTCATTGTTTTGCAGGGGC
OVP1_ F	GCTGGCAAGGACTTTGAG
OVP1 R	CCCTGCCAACTTGAGGAGGTC
FMDV2B_F	ATGCAGGAGGACATGTCAAC
FMDV2B_R	TTGATGTCACGTGCTTTGAG
FMDV 5′UTR_F	CTGTTGCTTCGTAGCGGAGC
FMDV 5′UTR_R	TCGCGTGTTACCTCGGGGTACC

## Data Availability

No new data was created.
